# Separation of unsaturated C18 fatty acids using perfluorinated‐micellar electrokinetic chromatography: I. Optimization and separation process

**DOI:** 10.1002/elps.202200151

**Published:** 2022-11-24

**Authors:** Hai Yen Ta, Lucie Perquis, Stéphane Balayssac, Christophe Déjugnat, Alexandre Wodrinski, Fabrice Collin, Véronique Gilard, François Couderc

**Affiliations:** ^1^ Laboratoire des IMRCP Université de Toulouse, CNRS UMR 5623, Université Toulouse III – Paul Sabatier Toulouse France

**Keywords:** ammonium perfluorooctanoate, fatty acids, micellar electrokinetic chromatography, self‐assembly, surfactant

## Abstract

Ammonium perfluorooctanoate (APFOA) was used as a surfactant for the separation of free unsaturated C18 fatty acids by micellar electrokinetic chromatography. A simple background electrolyte of 50 mM APFOA water/methanol (90:10, v/v) at pH = 10 enabled the repeatable separation of oleic acid, elaidic acid, linoleic acid, and alpha‐linolenic acid in less than 20 min. Separation conditions were optimized regarding various parameters (organic solvent, counterion, APFOA concentration, and pH). Because the repulsive interactions between fluorocarbon chains and hydrogenated chains are known to lead to segregation and phase separation, the choice of perfluorinated micelles to separate such perhydrogenated long‐chain acids could appear astonishing. Therefore, the critical micelle concentration, the charge density, and the mobility of the micelles have been determined, resulting in a first description of the separation process.

**Abbreviations**
ALA: alpha‐linolenic acid
APFOA: ammonium perfluorooctanoate
EA: elaidic acid
FAs: fatty acids
LA: linoleic acid
OA: oleic acid
PFOA: perfluorooctanoic acid
TFE: trifluoroethanol

## 1 INTRODUCTION

Fatty acids (FAs) represent one of the major lipid classes and are involved in many biological processes. Among the analytical techniques suitable for the separation of FAs, gas chromatography (GC) is the mainly used one. GC‐based protocols have been optimized very well, especially for the separation of FA methyl esters, and several applications have been described in the literature [1, 2, 3]. Capillary electrophoresis (CE) is another leading technique, now well described for the separation of free FAs [4, 5]. In most of the reported separation conditions, the use of sodium dodecyl sulfate (SDS) is necessary, in combination with organic solvents or cyclodextrins. This ensures a good solubilization of FAs, which are poorly water‐soluble and can themselves form micelles.

Micellar electrokinetic chromatography (MEKC) can be used for the separation of FAs [6, 7, 8], including detection in real matrixes [9, 10, 11], as well as nonaqueous capillary electrophoresis [12]. In the latter case, the separation is performed using organic solvents (acetonitrile, methanol, and isopropanol) in the presence of aqueous ammonia or ammonium acetate. These volatile electrolytes have the advantage to be useable when CE is coupled to electrospray ionization mass spectrometry (in CE/electrospray ionization [ESI]–mass spectrometry [MS] experiments), whereas MEKC using SDS is not compatible with ESI–MS due to its low volatility. To overcome this major drawback and enable MS detection, volatile surfactants have to be used in replacement of SDS.

Two decades ago, ammonium perfluorooctanoate (APFOA) has been used as a surfactant in MEKC experiments [13]. It was found to be a compatible volatile surfactant for MEKC–ESI/MS analysis of polar compounds, such as *N*‐methylcarbamates [14, 15], charged and uncharged phenyl compounds (more or less hydrophobic) [13], pyrene and benzopyrene [16], benzimidazoles [17], amino acids [18, 19], and so on. However, its use for FAs separation in MEKC–ESI/MS has not been reported so far. Moreover, the physical and chemical characteristics of the resulting micelles in the background electrolyte (BGE) have not been characterized neither.

The repulsive interactions between fluorocarbon chains and hydrogenated chains are known to lead to segregation and phase separation [20], due to severe nonidealities. The mixing of SDS and APFOA was found to result in demixing into two kinds of micelles with either a high‐level content of SDS or APFOA [21]. This is still under debate as another hypothesis about this kind of mixture is that it would form mixed micelles [22]. However, it seems that small amounts of hydrogenated surfactants can be incorporated into APFOA‐rich micelles, and small amounts of fluorinated surfactants into hydrogenated surfactant‐based micelles [23]. In this context, could APFOA—used as volatile surfactant—be a good candidate to separate FAs, keeping in mind that it could be later be compatible with MS analysis?

As a proof of concept, the latter possibility was positively examined for the separation of low concentrations of fatty acids by APFOA MEKC, keeping simple UV–Vis detection as a first step. Thus, APFOA micelles were used to separate four free unsaturated FAs, namely, oleic acid (OA), elaidic acid (EA), linoleic acid (LA), and alpha‐linolenic acid (ALA). The interactions underlying the separation of these hydrogenated compounds in the perfluorinated medium were studied, and a first approach of the separation mechanism was proposed. This manuscript will first present the optimization of CE separation. Then, the measurement of the mobility of the APFOA micelles was performed by using a micelle marker we synthesized. The CE instrument was also used to determine the cmc (critical micelle concentration), which was confirmed by nuclear magnetic resonance (NMR) experiments. Based on the residence time of FAs and geometric considerations, we propose a preliminary separation mechanism of FAs by APFOA micelles.

## 2 MATERIALS AND METHODS

### 2.1 Chemicals and reagents

Perfluorooctanoic acid (96%, PFOA), FAs (≥99%, including OA, EA, LA, and ALA), 2,2,2‐trifluoroethanol (TFE), phenylalanine (99%), sodium hydroxide, ammonium hydroxide, methanol, ethanol, and acetonitrile (HPLC grade solvents) were purchased from Sigma‐Aldrich (Saint‐Quentin‐Fallavier, France). Perfluorooctanoyl chloride (97%) was supplied from Fisher Scientific‐Alfa Aesar (Illkirch, France). Deuterated solvents were supplied from Eurisotop (Saint‐Aubin, France).

### 2.2 Preparation of samples and buffers

Stock solutions of separation buffer—the APFOA micellar solutions—were prepared by weighting and dissolving the appropriate amounts of PFOA in aqueous ammonium hydroxide (NH_4_OH). The concentration ratios *R*, defined as *R* = [NH_4_OH]/[PFOA], were *R* = 3.4 or *R* = 5.1. As a consequence, [NH_4_OH] was always proportionally related to [PFOA], depending on the *R* value. The addition of NH_4_OH to increase pH almost did not change the ionic strength because excess NH_4_OH remained mostly in a neutral form close to its p*K*a (9.2). The formation of NH_4_^+^ cations only came from the reaction between NH_4_OH and PFOA, maintaining [NH_4_^+^] equal to the initial [PFOA]. The pH of these stock solutions were measured using a SevenCompact S220 pH‐meter (Mettler–Toledo, Viroflay, France). The pH values were 9.5 and 10 for *R* = 3.4 and *R* = 5.1, respectively. The working BGEs used for optimizing the method were prepared by diluting 100 mM APFOA aqueous stock solutions with water and organic modifiers (i.e., MeOH, EtOH, or ACN) to get the desired final APFOA concentration and organic solvent content. As an illustrative example, the BGE composed of 50 mM APFOA along with 10% MeOH at pH 10 was prepared by mixing the 100 mM APFOA aqueous stock solution (pH = 10, *R* = 5.1) with water and MeOH in the ratio 50–40–10 (volume fractions). FAs stock solutions with a concentrations of 30 mM were prepared in methanol and kept at 5°C before use. The injection buffers containing the samples were prepared in 23 mM APFOA water/MeOH (47:53, v/v) to allow the solubilization of the four FAs (1 mM).

### 2.3 CE separation parameters

CE analysis was performed on an Agilent Technologies CE7100 system (Waldbronn, Germany). Separation of FAs was carried out on a 50 µm id × 365 µm od × 51 cm effective length (59 cm total length) bare‐fused silica capillary (Picometrics—Adelis Technologies, Labège, France).

New capillaries were rinsed with 1 M NaOH for 60 min, water (HPLC grade) for 30 min, and BGE for 15 min. Between two analyses, the capillary was rinsed with methanol for 3 min, 1 M NaOH for 3 min, water for 2 min, and BGE for 3 min. At the end of each day, the capillary was rinsed with methanol for 3 min, 1 M NaOH for 15 min, and water for 5 min. The pressure applied for the rinsing procedures was 950 mbar. Samples were hydrodynamically injected at 50 mbar for 5 s. All analyses were carried out at 25°C and at 25 kV for 30 min; the wavelength of UV direct detection was set at 195 nm.

### 2.4 cmc Determination of APFOA

#### 2.4.1 CE method

Solutions, containing PFOA at 5, 10, 15, 20, 25, 30, 35, 40, 45, 50, 60, 70, 80, 90, and 100 mM, and 5.1‐fold more ammonia, were flushed for 10 min in the separation capillary, then a voltage of +25 kV was applied. The currents were recorded, and the curve *I^c^* = f([APFOA]), which represents the variation of *I^c^* as a function of APFOA concentration, was drawn as reported in [24]. We obtained a curve that allowed the calculation of the cmc. This curve shows two linear portions, the first one at low APFOA concentrations as described in the following equation:

(1) $$\;I^{c}=a_{1}\cdot \left[\mathrm{APFOA}\right]$$

with *a_1_* = 6.67·10^−7^ A mM^−1^, and the second straight line at high APFOA concentrations as described below in the following equation:

(2) $$I^{c}=a_{2}\cdot \left[\mathrm{APFOA}\right]+b_{2}$$

with *a*_2_ = 4.5·10^−7^ A mM^−1^, *b*_2_ = 4.10·10^−6^ A.

[APFOA] is expressed in mM, and *I^c^* in A. At the intersection of these two lines, [APFOA] is equal to the cmc, which can then be obtained by combining Equations (1) and (2) as follows:

(3) $$\mathrm{cmc}=b_{2}\;/\left(a_{1}-a_{2}\right)$$

#### 2.4.2 NMR method

All solutions for NMR experiments were prepared with deuterated water as a solvent.

Fluorine‐19 NMR experiments at the different concentrations of APFOA (5, 7, 10, 15, 20, 25, 30, 40, 50, 60, 80, and 92 mM), including TFE as chemical shift reference (2 mM), were acquired on a Bruker Avance 400 spectrometer (Bruker Biospin AG, Fallanden, Switzerland) equipped with a 5 mm triple resonance probe (TXO) with ^19^F direct detection (376 MHz).

One‐dimensional (1D) ^19^F pulse sequence with power‐gated decoupling for ^1^H (zgpg in Bruker library) was used at 25°C with the following parameters: an acquisition time of 1.74 s with 256 K data points and a spectral width of 200 ppm, a relaxation delay of 2 s, and 16 scans.

Diffusion NMR spectra were recorded with a classical stimulated echo NMR experiment using bipolar gradients (stebpgp1s in Bruker library). The acquisition parameters were as follows: 64k data points, acquisition time 1.19 s, spectral width 80 ppm, number of scans 16, relaxation delay 3 s, pulse field gradient length 3.6 ms and recovery delay 1 ms, and diffusion time 200 ms. The spoiler gradients were 0.6 ms long. Sixteen experiments were recorded with a linear gradient sampling from 5% to 95% of the maximum gradient strength.

All NMR data were processed using the TOPSPIN 4.0.8 software with one level of zero‐filling and an exponential line‐broadening function of 0.5 Hz. Spectra were calibrated at −76.96 ppm on the CF_3_ signal of TFE. The data sets of diffusion NMR experiments were processed with TOPSPIN software and transferred to the Origin 9.1 software. The exponential fit tool was then used to determine the values of the self‐diffusion coefficient (*D*).

^19^F NMR assignments of APFOA were given at 50 mM (CE conditions) as previously reported by Liu and Goddard [25]: F2 (−117.91 ppm, tt, 3.0 and 12.8 Hz, –C**F_2_**–COOH), F3 (−122.58 ppm, m, –**CF_2_**–CF_2_–COOH), F4 (−122.86 ppm, m, –**CF_2_**–CF_2_–CF_2_–COOH), F5 (−123.49 ppm, m, –**CF_2_**–CF_2_–CF_2_–CF_3_), F6 (−123.65 ppm, m, –**CF_2_**–CF_2_–CF_3_), F7 (−127.23 ppm, m, –**CF_2_**–CF_3_), and F8 (−82.34 ppm, tt, 2.2 and 10.1 Hz, –CF_2_–**CF_3_**).

The determination of the cmc was performed as described by Xing et al. [26] using the following equation based on the theory of pseudo‐phase transition model:

(4) $$\Delta \delta =\frac{\mathrm{cmc}}{C}\left(\delta_{mon}-\delta_{mic}\right)+\delta_{mic}-\delta_{mon}$$

where Δ*δ* is the chemical shift variation of selected ^19^F NMR signals in each solution compared to the solution at a concentration below the cmc (5 mM in the present study), *δ_mon_* and *δ_mic_* are the chemical shifts of monomers and micelles, respectively, and *C* is the concentration of the solution analyzed. The cmc can be obtained based on the curve Δ*δ* = f(1/*C*). A constant line function is observed at concentrations below the cmc and become a linear function of 1/*C* in the micellar region. The intercept between these two straight lines defines the cmc.

### 2.5 Synthesis of the micelle marker, the *N*‐perfluorooctanoyl phenylalanine

**FIGURE 1:**
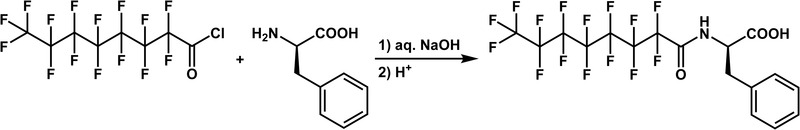
Synthesis of the micelle marker by direct acylation of phenylalanine

The target compound was prepared via a classical Schotten–Baumann acylation (Figure 1). Phenylalanine (0.215 g, 1.3 mmol) was dissolved in 2 ml aqueous NaOH (0.2 M) and placed at 0°C. Perfluorooctanoyl chloride (0.433 g, 1 mmol) was added dropwise followed by additional 2 ml aqueous NaOH (0.2 M). The mixture was stirred for 1 h at 0°C then for 4 h at room temperature. The white suspension was acidified till pH = 1 using aqueous 6 M HCl. After filtration and thorough washes with water then methanol, the resulting solid was dried under vacuum affording a white powder (0.157 g, 28% yield). NMR characterizations were in accordance with literature [27, 28].

High‐resolution MS (HRMS) analysis was performed by ESI coupled to a time‐of‐flight (ToF) analyzer, in the negative mode (Xevo G2 QToF, Waters). Mass obtained for C_17_H_9_NO_3_F_15_ (M–H)^−^ are: *m*/*z* 560.0343 (calculated) and *m*/*z* 560.0347 (experimental) (+0.7 ppm).

^1^H NMR (DMSO‐d_6_, 400 MHz): (9.76 ppm, d, ^3^ *J*_HH_ = 8.5 Hz, 1*H*, NH), (7.34–7.16 ppm, m, 5*H*, CH_arom_), (4.57 ppm, ddd, ^3^ *J*_HH_ = 8.5 Hz, ^3^ *J*_HH_ = 4 Hz, ^3^ *J*_HH_ = 12 Hz, 1*H*, ^α^CH), (3.23 ppm, dd, ^2^ *J*_HH_ = 14 Hz, ^3^ *J*_HH_ = 4 Hz, 1*H*, 1/2xCH_2_), and (3.01 ppm, dd, ^2^ *J*_HH_ = 14 Hz, ^3^ *J*_HH_ = 12 Hz, 1*H*, 1/2xCH_2_).

^13^C NMR (DMSO‐d_6_, 100 MHz): (171.3 ppm, –COOH), (156.6 ppm, –CONH), (137.3 ppm, C_q,arom_), (128.9 ppm, CH_arom_), (128.1 ppm, CH_arom_), (126.4 ppm, CH_arom_), (116.6 ppm, –CF_3_), (110.2 ppm, –CF_2_), (110.2 ppm, –CF_2_), (110.0 ppm, –CF_2_), (109.7 ppm, –CF_2_), (108.5 ppm, –CF_2_), (107.8 ppm, –CF_2_), (54.2 ppm, –CH), and (35.4 ppm, –CH_2_).

^19^F NMR (DMSO‐d_6_, 376 MHz): (−80.3 ppm, 3F), (−118.6 ppm, 2F), (−121.6 ppm, 2F), (−122.0 ppm, 2F), (−122.6 ppm, 4F), and (−125.9 ppm, 2F).

## 3 RESULTS AND DISCUSSION

### 3.1 Optimization of MEKC conditions for the separation of mono‐, di‐, and tri‐unsaturated free C18 FAs

**FIGURE 2:**
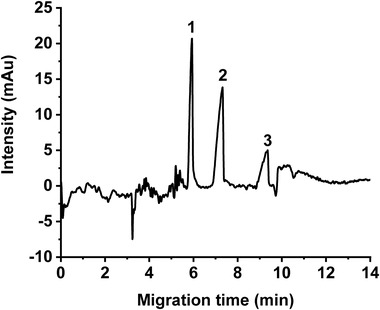
Separation of the three *cis*‐isomers free fatty acids (FAs) C18: (1) alpha‐linolenic acid (ALA), (2) linoleic acid (LA), and (3) oleic acid (OA). Capillary electrophoresis (CE) conditions: fused silica capillary (id: 50 µm, *L_tot_*: 59 cm, *L_eff_*: 51 cm), background electrolyte (BGE) consisting of 50 mM ammonium perfluorooctanoate (APFOA) at pH 9.5, [NH_4_OH]/[PFOA] = 3.4; samples injected at 50 mbar for 5 s, +25 kV, 25°C, detection at 195 nm. FA injected concentration: 1 mM

A standard mixture of three *cis*‐isomers free C18 FAs (OA, LA, and ALA) was first analyzed by using a BGE of 50 mM APFOA at pH 9.5. At this initial chosen pH, the ratio between concentrations of ammonium hydroxide and PFOA ([NH_4_OH]/[PFOA]) was *R* = 3.4. Peak assignation was realized by comparison to reference migration time of each acid, as individually injected. The obtained electropherogram exhibited a noisy baseline due to a strong absorption of the APFOA medium at 195 nm (Figure 2). In addition, all FA peaks were asymmetric. In order to gain in separation performance, the composition of the BGE was optimized (nature and amount of the added organic solvent, APFOA concentration, [NH_4_OH]/[PFOA] ratio), along with the presence of methanol in the sample composition.

#### 3.1.1 Influence of the organic solvent added in the BGE

**FIGURE 3:**
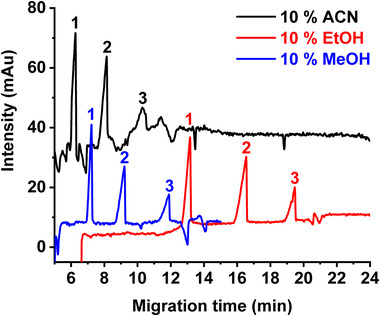
Effect of organic solvents on the separation of three unsaturated free fatty acids (FAs) C18: (1) alpha‐linolenic acid—ALA, (2) linoleic acid—LA, and (3) oleic acid—OA. ACN: acetonitrile, EtOH: ethanol, and MeOH: methanol. Capillary electrophoresis (CE) conditions: fused silica capillary (id: 50 µm, *L_tot_*: 59 cm, *L_eff_*: 51 cm). Background electrolyte (BGE): 50 mM ammonium perfluorooctanoate (APFOA) at pH 9.5 water/organic solvent (90:10, v/v), [NH_4_OH]/[PFOA] = 3.4. Samples injected at 50 mbar for 5 s. +25 kV, 25°C, and *λ*: 195 nm. FAs injected concentration: 1 mM

The presence of an organic solvent usually helps dissolving FAs and often allows better separation. It also has an influence on the viscosity of the BGEs and consequently affects the separation times. The addition of 10% (v/v) acetonitrile (ACN), methanol, or ethanol in the BGE was tested. The corresponding viscosities were reported to be 0.96, 1.07, and 1.20 cP for water/ACN, water/MeOH, and water/EtOH, respectively [29, 30]. The obtained electropherograms (Figure 3) showed that acetonitrile led to the fastest separation of FAs but also to the noisiest baseline. Electropherograms with more stable baselines were obtained with methanol and ethanol, and the three FAs were well separated. However, the presences of ethanol in the BGE lead to the longest migration times. Thus, methanol was selected as it allows obtaining an efficient separation in the shortest migration time.

The methanol content in the BGE was then optimized. By increasing it from 10%, 15% to 20% (v/v), the viscosity of the separation medium was increased, leading to longer migration times and peak broadening. At 20% (v/v) methanol in the BGE, we did not observe any peak for OA (the most slowly migrating FA) after 70 min of analysis (data not shown). Thus, the methanol content was set at 10% (v/v) for the next optimizations.

#### 3.1.2 Effect of the counter ion

**FIGURE 4:**
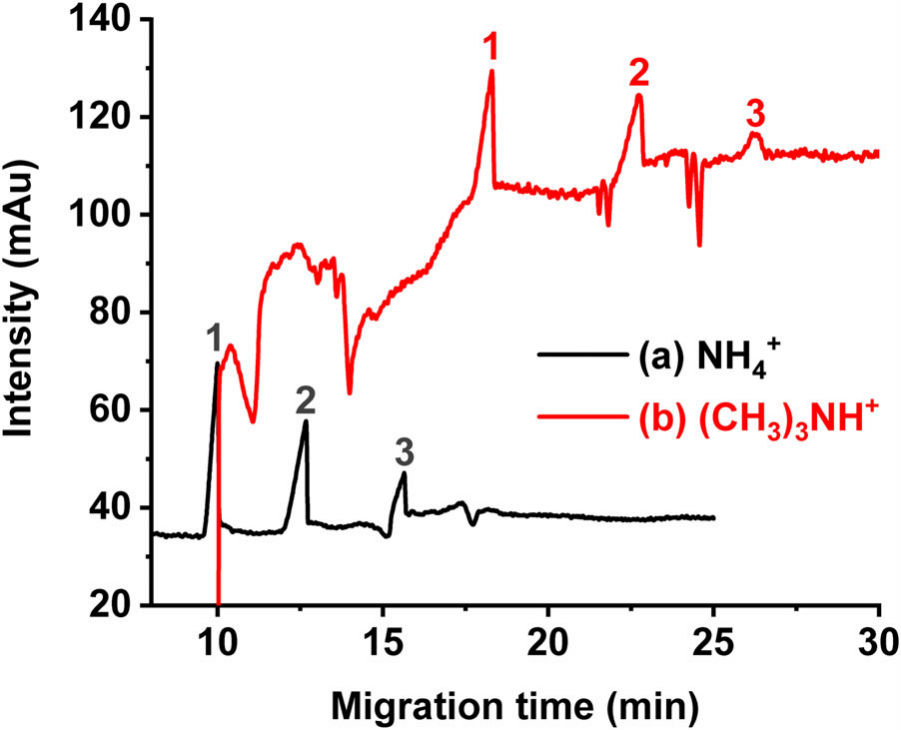
Effect of counterion on the separation of three unsaturated free fatty acids (FAs) C18: (1) alpha‐linolenic acid—ALA, (2) linoleic acid—LA, and (3) oleic acid—OA. Capillary electrophoresis (CE) conditions: fused silica capillary (id: 50 µm, *L_tot_*: 59 cm, *L_eff_*: 51 cm). (A) Background electrolyte (BGE): 50 mM ammonium perfluorooctanoate pH 9.5/MeOH (90:10, v/v) and (B) BGE: 50 mM trimethylammonium perfluorooctanoate pH 9.5 water/MeOH (90:10, v/v). Samples injected at 50 mbar for 5 s. +25 kV, 25°C, *λ*: 195 nm. FAs injected concentration: 1 mM

It is well known that the micelle properties not only depend on the nature of the surfactant but also on the type of counterion present at the micellar surface. In this study, we used ammonia or trimethylamine to adjust the pH of PFOA solutions, resulting in the presence of NH_4_^+^ or (CH_3_)_3_NH^+^ as counterions, respectively. The results showed that, as compared to NH_4_^+^, longer migration times were obtained for ALA, LA, and OA in the presence of (CH_3_)_3_NH^+^ and that the corresponding electropherogram presented a very noisy baseline (Figure 4), whereas the peak symmetries were not improved. Consequently, the ammonium ion NH_4_^+^ was selected as counterion in the separation electrolyte.

#### 3.1.3 Effects of APFOA concentration and NH_4_OH/PFOA ratio

**FIGURE 5:**
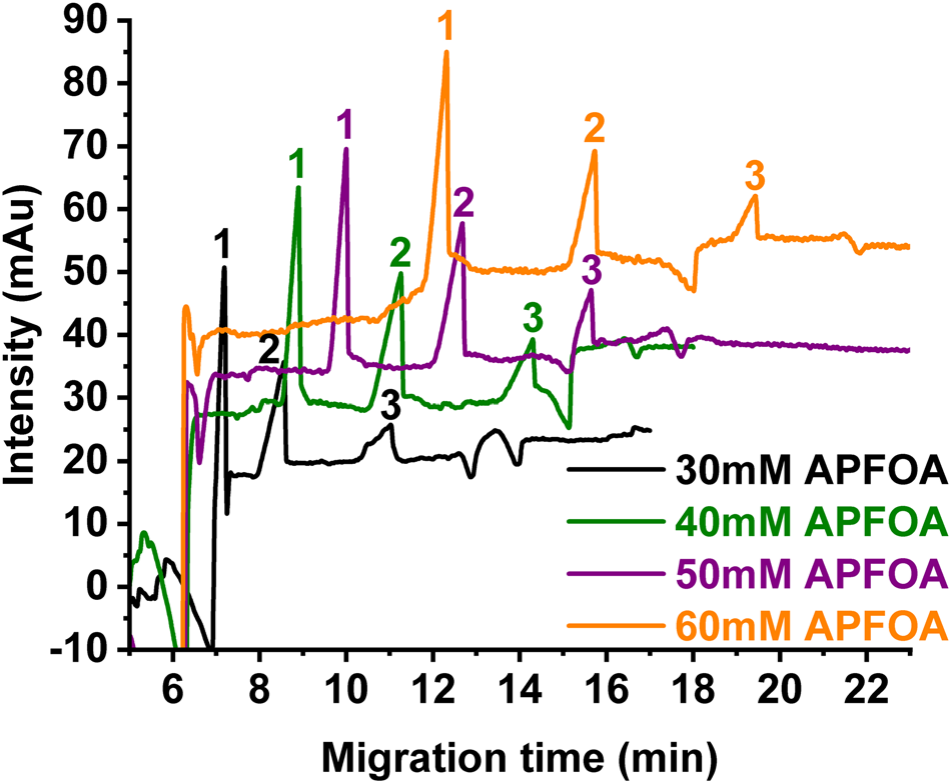
Effect of ammonium perfluorooctanoate (APFOA) concentration on the separation of three unsaturated free fatty acids (FAs) C18: (1) alpha‐linolenic acid—ALA, (2) linoleic acid—LA, and (3) oleic acid—OA. Capillary electrophoresis (CE) conditions: fused silica capillary (id: 50 µm, *L_tot_*: 59 cm, *L_eff_*: 51 cm). Background electrolyte (BGE): 50 mM APFOA at pH 9.5 water/methanol (90:10, v/v), [NH_4_OH]/[PFOA] = 3.4. Samples injected at 50 mbar for 5 s. + 25 kV, 25°C, *λ*: 195 nm. FAs injected concentration: 1 mM

The influence of APFOA concentration on the separation of OA, LA, and ALA was also studied (Figure 5). APFOA concentrations ranging from 20 to 60 mM were tested ([NH_4_OH] = 68–204 mM at *R* = 3.4), in the presence of 10% MeOH in the BGE. At 20 mM APFOA, the three FAs cannot be separated, whereas the separation was achieved at 25 mM APFOA but the peak resolutions were too low (data are not shown for those two concentrations). At such low concentrations, just above the cmc, only very small quantities of APFOA micelles are formed in the BGE. In contrast, the three FAs were well separated at 30 mM APFOA and above (Figure 5). Increasing APFOA concentration improved peak resolutions but also led to increased migration times. Moreover, for the highest surfactant concentration, some problems appeared such as unstable current or clogging of the capillary. With a view to analyzing more complex samples containing ALA, LA, and OA, the quality of separation was considered the most relevant criterion to choose the BGE composition. The quality of separation was evaluated considering peak visibility and separation as well as baseline quality. Compared to 40 mM, the baseline is more stable at 50 mM and the peak 3 is more visible. Therefore, 50 mM APFOA along with 10% MeOH was selected as BGE composition.

**FIGURE 6:**
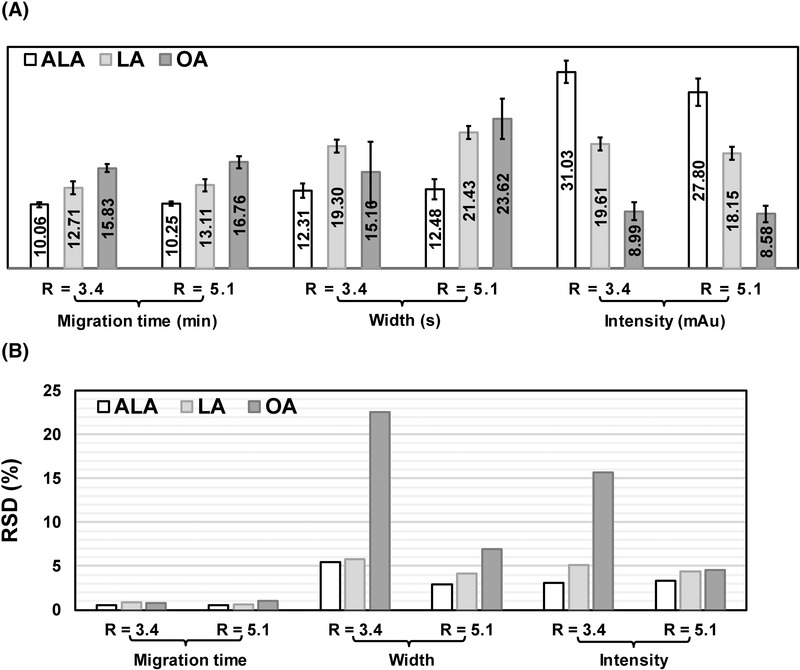
(A) Effects of *R* = [NH_4_OH]/[PFOA] on the migration times, peak widths, and intensities of three fatty acids (FAs): alpha‐linolenic acid (ALA), linoleic acid (LA), and oleic acid (OA). A triplicate injection has been performed for each sample. All reported values were averaged. (B) Effect of the *R* ratio on the interdays repeatability (*n* = 4) of the previous parameters, reported as relative standard deviation (RSD,%)

The pH also plays an important role in the separation buffer because it defines the ionization forms of species in solution and thus affects their electrophoretic mobilities. The separation of FAs often requires a buffer at pH > 9 to ensure a strong electroosmotic flow (EOF). In our case, working at high pH also helped to maintain the buffering efficiency of the ammonia solution (pKa ∼ 9.2). The influence of the ammonia concentration or the pH was then evaluated. Therefore, the initial ratio *R* = [NH_4_OH]/[PFOA] = 3.4 (pH = 9.5) was then increased to 5.1 (pH = 10). The separation performances of the three FAs were compared in these two conditions. Different parameters, including migration times, peak widths, and intensities where under focus, and the repeatability of the analysis was evaluated. The results in Figure 6A (left) showed that increasing 1.5 times the ammonia concentration in the BGE led to slightly longer migration times of FAs (2% for ALA, 4% for LA, and 6% for OA). At *R* = 3.4, FA peak widths were reduced, and peak intensities were slightly higher than at *R* = 5.1 (Figure 6A, center and right). In contrast, higher ammonia concentration (*R* = 5.1) led to better repeatabilities of peak widths and intensities, in particular for OA, for which relative standard deviations were 7% and 5%, respectively (Figure 6B), instead of 23.5% and 15.5%, respectively, at *R* = 3.4. Therefore, the [NH_4_OH]/[PFOA] ratio at 5.1 has finally been preferred and selected for the separation of these three FAs.

#### 3.1.4 Optimization of the methanol content in the sample (injection buffer)

**FIGURE 7:**
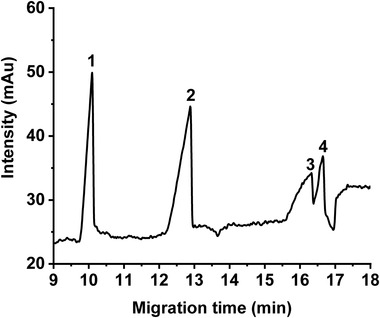
Separation of four unsaturated free fatty acids (FAs) C18: (1) alpha‐linolenic acid—ALA, (2) linoleic acid—LA, (3) oleic acid—OA, and (4) elaidic acid—EA. Capillary electrophoresis (CE) conditions: fused silica capillary (id: 50 µm, *L_tot_*: 59 cm, *L_eff_*: 51 cm). Background electrolyte (BGE): 50 mM ammonium perfluorooctanoate (APFOA) at pH = 10 water/MeOH (90:10, v/v), [NH_4_OH]/[PFOA] = 5.1. Samples injected at 50 mbar for 5 s. +25 kV, 25°C, *λ*: 195 nm. Concentration of each FA: 1 mM

The methanol content in the sample was optimized to enable analysis of both *cis*‐ and *trans*‐isomers of FAs. In a first approach, the sample preparation of FAs consisted in their dilution in the BGE. The three *cis*‐isomers ALA, LA, and OA were found to easily dissolve in the BGE consisting of 50 mM APFOA with 10% (v/v) methanol. However, in the same conditions, the *trans*‐isomer of OA, namely EA, was found to form an opaque solution, resulting in an electropherogram with unstable baseline. Therefore, the BGE was gradually diluted with methanol to get an injection buffer containing EA giving satisfying CE analysis. Considering solubility, peak widths, and intensities, the optimal amount was found to be 53% (v/v) methanol. This composition was then used for all samples. Figure 7 presents the CE analysis a sample containing a mixture of ALA, LA, OA, and EA (1 mM each), using the simple (previously optimized) BGE consisting of 50 mM APFOA ([NH_4_OH]/[PFOA] = 5.1, pH = 10) with 10% (v/v) methanol. It shows the effective separation of these FAs differing by the number of unsaturations (ALA, LA, and OA) and the *cis*/*trans* configuration (OA and EA, with resolution peak = 1.6).

Cyclodextrins and derivatives are often added in the BGE to improve the separation of isomer mixtures. Thus, we tried adding 5–15 mM β‐cyclodextrin in the BGE to get a better OA versus EA separation. However, in these conditions, the resolution of those two latter peaks was not improved.

Despite the optimization of all abovementioned parameters, we always observed asymmetric peaks. Only the dilution of FA samples improved peak symmetry. However, APFOA has a high UV absorption which severely limits FA detection upon dilution and imposes working at high analyte concentration (1 mM) in the sample.

### 3.2 Characterization of APFOA micelles (50 mM APFOA, *R* = 5.1 in 10% methanol v/v)

The mechanism allowing the separation among FAs using APFOA micelles remains unclear: How the poor interactions between perfluorinated chains and perhydrogenated chains could assist the separation of these unsaturated C18 acids? To understand this point, we initiated the characterization of APFOA micelles. We first conducted experiments using the CE instrument to determine the cmc and the charge density of the micelles. Then, we also determined the mobility of the perfluorinated micelles by introducing a PFOA analogue marker, namely, the *N*‐perfluorooctanoyl phenylalanine.

#### 3.2.1 Determination of the cmc of APFOA

**FIGURE 8:**
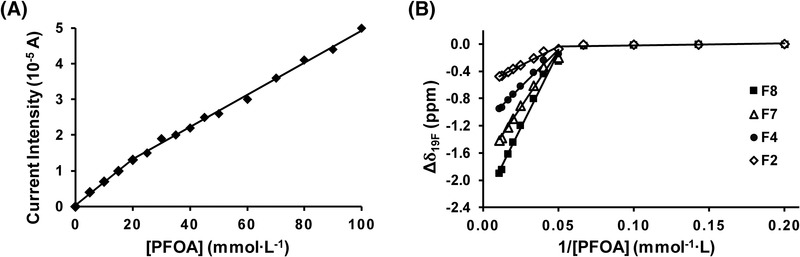
Determination of perfluorooctanoic acid (PFOA) critical micellar concentration in the presence of 10% (v/v) methanol by (A) capillary electrophoresis (CE) and (B) nuclear magnetic resonance (NMR). (A) Current intensity in the capillary as a function of PFOA concentration. CE conditions: fused silica capillary (id: 50 µm, *L_tot_*: 59 cm, *L_eff_*: 51 cm). Background electrolytes (BGEs): *x* mM ammonium perfluorooctanoate (APFOA) at pH 9.5 water/methanol (90:10, v/v), *x* = 5–50 mM, [NH_4_OH]/[PFOA] = 5.1. +25 kV, 25°C, *λ*: 195 nm. (B) Chemical shift changes of fluorine (Δ*δ*_19F_) measured at 376 MHz, 25°C as a function of the reciprocal of PFOA concentration. F2, F4, F7, and F8 refer to ^19^F NMR assignments of APFOA as reported in the experimental section

The cmc of APFOA in the presence of 10% (v/v) methanol and at *R* = 5.1 ([NH_4_OH]/[PFOA]) was determined. The cmc is the minimal concentration at which APFOA micelles start to form, and above which the concentration of free APFOA (C^RCOO−^) remains constant and equal to the cmc. The formation of micelles induces a change in the conductivity of the medium. The conductivities of APFOA solutions were obtainable via the current intensities in the capillary, which were plotted as a function of PFOA concentration (Figure 8A). The curve consisted in two linear parts with different slopes: The cmc was obtained at the intersection of these two parts.

As proposed by Cifuentes et al. [24] we can measure the cmc of the APFOA in such solution (Figure 8A). Here, the cmc was determined to be 19 ± 0.9 mM. The confirmation of the cmc value was performed by NMR experiments by following the chemical shift variations of ^19^F, and by plotting them as a function of the reciprocal PFOA concentration (Figure 8B). The resulting curve consisted in two linear parts, the second having an almost zero slope. The cmc was obtained at the intersection of these two parts and found to be 19 ± 0.9 mM. Thus, the NMR data also confirmed this value.

#### 3.2.2 Degree of counterion dissociation of the perfluorinated micelles

The degree of counterion dissociation can be determined as the ratio of the slopes above cmc (a_2_) and below cmc (a_1_) in the *I* = f(*C^t^*) representation (Figure 8A) [31], as described in the experimental section. This ratio gives the degree of counterion dissociation, *β*, and subsequently the degree of counterion association *α*: *α* = (1 − *β*). In our case, *β* = 0.68, which is a very high value as compared to the one obtained for SDS micelles (*β* = 0.34 [27], or *β* = 0.28 [32]). In another study, Kancharla et al. have found a *β* value of 0.47, in the absence of MeOH in the BGE. The value was found to greatly increase in the presence of urea [33]. Short‐chain alcohols and urea have similar effects on the cmc of APFOA as its value decreases when these compounds are added to the APFOA solutions [33, 34]. Therefore, both MeOH and urea could have a similar effect on the counterion dissociation: The presence of MeOH in the BGE could explain the high *β*‐value observed in our experimental conditions. Such a significant value makes the micelles clearly negatively charged and not neutral.

#### 3.2.3 Measurements of the mobilities of the micelles and the free APFOA

To measure the mobility of the APFOA micelles (*µ^mic^*), an aromatic analog of PFOA was synthesized by grafting PFOA on phenylalanine via an amide link. The obtained compound, which will have an important affinity for APFOA micelles, will allow measuring the migration time of these aggregates by UV‐detection at 260 nm (absorption of phenylalanine). We obtained a migration time of 146 ± 5 min (*n* = 3), which is much higher than the one of the measured EOF, *t_EOF_* = 3.25 min (injection of water/MeOH 47/53, v/v). In MEKC, the negatively charged micelles normally have a negative mobility compared to the EOF. Moreover, the velocity of the micelles in absolute value is very close to the one of the EOF: the calculated values are *µ^EOF^* = 61.7·10^−9^ m^2^ V^−1^ s^−1^ and *µ^mic^* = −60.4·10^−9^ m^2^ V^−1^ s^−1^. Thus, there is almost no movement of the micelles inside the capillary and the separation of FAs in these conditions depends on their interaction and residence time in the micelles.

#### 3.2.4 Capacity factor (*k′*) and residence time (RT) of the FAs in the micelles

To better understand the mechanism underlying the separation of FAs in our experimental conditions, their capacity factor and their residence time in the micelles have been studied. Terabe et al. [35, 36] proposed the measurement of *k′* to appreciate the interaction between the solutes (here the FAs) and the micelles, for neutral species. Khaledi et al. completed this study for acidic solutes [37]. *k′* is defined as

(5) $$k^{\prime }=n_{mic}\;/\;n_{aq}=\left(\mu^{ag}-\mu^{FA}\right)\;/\left(\mu^{mic}-\mu^{ag}\right)$$

where *n_mic_* and *n_aq_* are the molar quantities of the solute in the micelles and in the aqueous media, respectively, *µ^ag^* is the mobility of the FAs using a micellar medium (i.e., corresponding to the fatty acid migration time), whereas *µ^FA^* is their mobility in the absence of micelles. *µ^FA^* is not easy to determine because FAs are not soluble in 10% methanol and 255 mM ammonia. *µ^FA^* was thus estimated at the cmc of APFOA, considering that, at the cmc, the concentration of micelles is very low and can be neglected. In these conditions, we found an average value of *µ^FA^* = −20.8·10^−9^ m^2^ V^−1^ s^−1^.

The residence time *RT_mic_* of the solute in the micelle is defined as

(6) $$RT_{mic}=t_{r}\cdot k^{\prime }/\left(1+k^{\prime }\right)$$

where *t_r_* and *k′* are the migration time and the capacity factor, respectively. Table 1 reports the values obtained for the migration time, capacity factor, and residence time for the three FAs ALA, LA, and OA.

**TABLE 1:** Micellar electrokinetic chromatography (MEKC) parameters of the perfluorinated micelles

	*t*_mc_ 146.0 min, *t*_0_ 3.25 min, [APFOA] 50 mM		
	***t_r_* (min)**	***k'***	*RT_mic_* (min)
**ALA**	10.8	1.3	6.1
**LA**	13.2	1.9	8.6
**OA**	16.9	2.8	12.4

*Note*: Migration time *t*_r_, capacity factor *k*′, residence time in the micelles *RTmic*, of the three fatty acids (FAs) in the perfluorinated micelles.

Abbreviations: ALA, alpha‐linolenic acid; APFOA, ammonium perfluorooctanoate; LA, linoleic acid; OA, oleic acid.

The residence times of FAs are ranging from 6.1 to 12.4 min in the micelles, suggesting that FAs spend 56%–73% of their migration time into the micelles. Such high values were not expected as interactions are usually not favored between perhydrogenated molecules and perfluorinated micelles. In addition, the residence time decreases as the number of unsaturations of the FAs increases: OA (1 unsaturation) is more prone to insert into micelles than ALA (3 unsaturations). As a consequence, reminding that the micelles almost do not move inside the capillary, FAs that reside less in these micelles will migrate first in the order ALA > LA > OA. In this system, the separation mechanism originates directly from the interactions between the analytes and the micelles. From these observations, we can suggest a separation process similar to what is usually observed in the case of SDS used in MEKC, which remains quite surprising considering the repulsive interactions between perhydrogenated and perfluorinated chains. Complementary structural analysis at the mesoscale will provide more in‐depth characterization of the separation mechanism [38].

## 4 CONCLUDING REMARKS

In this article, we have shown that perfluorinated micelles of APFOA in the presence of 10% methanol and a ratio [NH_4_OH]/[PFOA] = 5.1 allow the separation of four unsaturated C18 FAs by MEKC. The relatively high electrophoretic mobility (*µ^mic^* = −60.4·10^−9^ m^2^ V^−1^ s^−1^) of the micelles is certainly due to the high degree of counterion dissociation of the perfluorinated micelles (*β* = 0.68). This mobility is much higher than the micelles of SDS (−22.2 m^2^ V^−1^ s^−1^) [35]. In the literature it is reported that the mixture of perfluorinated micelles with perhydrogenated ones exhibits severe non idealities [21]. However, the successful separation of the different FAs indicates that there is a specific interaction with the micelles, which constitute a pseudo‐chromatographic phase, because their displacements are very slow, that is, their electrophoretic mobilities are almost compensated by the EOF. Indeed, the residence times of FAs in the micelles are long, more than 56% of their full migration times. In this study, we show that it is possible to separate FAs thanks to their number of double bounds or the double bond position in the carbon chain. However, the characterization of the interactions between the FAs and the micelles is not accessible with CE. Therefore, we conducted other complementary studies involving scattering experiments that are described in a complementary article [38].

## CONFLICT OF INTEREST

The authors have declared no conflict of interest.

## Data Availability

The data that support the findings of this study are available from the corresponding author upon reasonable request.
